# A radiating Kerr black hole and Hawking radiation

**DOI:** 10.1016/j.heliyon.2020.e03336

**Published:** 2020-01-31

**Authors:** Yu-Ching Chou

**Affiliations:** Health 101 clinic, 1F., No. 97, Guling Street, Taipei City, Taiwan

**Keywords:** Astrophysics, Cosmology, Special relativity, Thermodynamics, Ellipsoid coordinate transformation, Hawking radiation, General relativity, Kerr metric, Radiating rotating black hole, Vaidya–Kerr metric

## Abstract

This study proposes an axisymmetric generalization of the Vaidya metric, namely the Vaidya–Kerr metric, to describe a radiating rotating black hole, and presents its Hawking radiation temperature. This study is an improved version of our previous research via ellipsoid coordinate transformation, and the Einstein field equations are solved concisely and intuitively by an orthogonal ansatz. The results demonstrate that the energy–momentum tensor of the derived radiating Kerr metric satisfies the energy-conservation law and is classified as a Petrov type II fluid, whereas the stationary Kerr metric is a Petrov type IV vacuum. The inner and outer event-horizon radii, the ergosphere radii, as well as the angular velocity at the event horizon are solved, and then, surface gravity, entropy, and Hawking radiation are derived. We estimate the Hawking-radiation temperature of the black holes with the angular momentum and the same mass of Pluto and the sun, as well as the supermassive black hole in the core of the M87 galaxy to be 9.42K, $6.08\times 10^{-8}$K, and $8.78\times 10^{-18}$K, respectively. Only the value of the rotating Pluto-mass black hole is slightly greater than the 3K cosmic microwave background radiation and may be detected by high-resolution tools in the future.

## 1 Introduction

The black hole solution of the four-dimensional spacetime Einstein–Maxwell equations of classical general relativity has the following physical characteristics: mass (*M*), electric charge (*Q*), and angular momentum (*J*). The static spherically symmetric solution is a Schwarzschild metric with mass as its only physical characteristic [1]. The static spherically symmetry solution with electric charge is the Reissner–Nördstrom metric [2], [3], and the axisymmetric generalization of the Schwarzschild metric with angular momentum is known as the Kerr metric [4]. The axisymmetric solution of the Reissner–Nördstrom metric has been generalized by incorporating angular momentum to the Kerr–Newman metric [5]. These four metrics are often referred to as the “black hole” exact solutions of general relativity.

In 1943, Vaidya proposed a radiating spherically symmetric solution [6]. The Vaidya metric, which was originally applied to radiating stars, can be regarded as the simplest generalization of the Schwarzschild metric. In 1974, Hawking applied quantum theory to determine that black holes emitted heat radiation [7]. Quantum theory predicts that black-hole mass will gradually evaporate through radiation; therefore, the black hole may have a fourth physical characteristic, i.e., temperature (*T*). Therefore, the Vaidya solution can be applied to black holes to study Hawking radiation. Moreover, celestial bodies present in the nature, which require the Vaidya–Kerr solution, always have rotational angular momentum and radiation, including radiating rotating stars and black holes.

Einstein's field equations are nonlinear differential equations; it is difficult to obtain accurate analytical solutions without using symmetry. The methods known for solving axisymmetric problems in the literature are as follows. First, the Newman–Janis algorithm (NJA) [8], [9], which usually requires Newman–Penrose formalism, is a commonly applied technique based on the use of complex null tetrads with ideas taken from 2-component spinors for general relativity [10], [11]. Second, the Papapetrou gauge and Ernst equations can also be applied to solve the axisymmetric Einstein's field equations [12], [13]. In addition, our previously conducted studies have demonstrated that the Kerr and Kerr–Newman metric can be derived from the orthogonal ansatz by applying an ellipsoidal-coordinate transformation [14], [15].

Recently, several radiating rotating solutions have been proposed. First, a study conducted by Ibohal discussed the axisymmetric Vaidya–Kerr metric, which admits nonperfect fluids [16]. Ghosh and Maharaj applied the Hayward black-hole solution as a seed metric to obtain a rotating radiating black hole without a singularity [17]. The aforementioned studies were based upon the Newman–Janis algorithm and Eddington–Finkelstein coordinates [18], [19]; they comprise a pair of coordinate systems, which are adapted to radial null geodesics for a Schwarzschild geometry.

A study conducted by Bohmer and Hogan proposed a different method for obtaining the Vaidya–Kerr spacetime [20]. They utilized a retarded u=t−r coordinate transformation to rewrite the non-static mass function and rotating parameter as m(u) and a(u), respectively in terms of the Kerr–Schild coordinates, which express the Kerr solution using Cartesian coordinates [21]. All results of these previously conducted studies involve radiating rotating black hole solutions.

This study aims to obtain a radiating Kerr metric via ellipsoid coordinate transformation, and to present the Hawking radiation temperature. Our derivation is a nonstationary generalization of our previous research [14], [15]; however, this paper presents an improved version of the orthogonal ansatz as well as a more concise and intuitive derivation method. The rest of this manuscript is organized as follows. Section 2 introduces the Vaidya and Kerr metrics. Section 3 introduces an ellipsoid coordinate transformation. Section 4 presents the derivation of the static Kerr metric. Section 5 presents the radiating Kerr black hole solution. Section 6 presents the physical properties of the Vaidya–Kerr metric. Section 7 presents the energy-momentum tensor. Section 8 presents the results and discussion. Section 9 concludes this paper. This paper sets c=G=1.

## 2 Vaidya and Kerr metrics

The Vaidya metric describes the external spacetime of spherically symmetric and non-rotating stars or black holes, which is either emitting or absorbing null dusts. It is a generalization of the simplest non-static Schwarzschild metric, and is expressed in Eddington coordinates (u,r,θ,ϕ), as follows

(1) $$ds^{2}=-\left(1-\frac{2m(u)}{r}\right)du^{2}+2\epsilon dudr+d\Omega^{2},(\epsilon =\pm 1)$$

where $d\Omega^{2}=d\theta^{2}+sin^{2}\theta d\phi^{2}$. ϵ=+1 represents the “advanced” or “ingoing” Vaidya metric, while ϵ=−1 represents the “retarded” or “outgoing” Vaidya metric. When mass function m(u)= constant *M*, the metric returns to the Schwarzschild metric. The Kerr metric, which is a generalization of the Schwarzschild metric, is another exact solution of general relativity. It can be used to describe the vacuum spacetime around a spherically symmetric celestial body. The Kerr metric in Boyer–Lindquist coordinates can be expressed as follows:

(2) $$ds^{2}=-\frac{\Delta_{K}}{\Sigma }{\left(dt-asin^{2}\theta d\phi \right)}^{2}+\frac{\Sigma }{\Delta_{K}}+\Sigma d\theta^{2}+\frac{sin^{2}\theta }{\Sigma }{\left[(r^{2}+a^{2})d\phi -adt\right]}^{2},$$

where $\Sigma =r^{2}+a^{2}cos^{2}\theta$, $\Delta_{K}=r^{2}-2Mr+a^{2}$.

The parameters *M*, *J*, and *a*, denote the constant mass, angular momentum, and rotation parameters, which are defined as a=J/cm, respectively.

## 3 Ellipsoid orthogonal coordinate transformations

To derive static axisymmetric solutions, one may start from a Minkowski spacetime, expressed in Cartesian form as

(3) $$ds^{2}=-dt^{2}+dx^{2}+dy^{2}+dz^{2}$$

We apply the following ellipsoid coordinate transformations to Eq. (3):

(4) $$x\to \;{\left(r^{2}+a^{2}\right)}^{1/2}sin\theta cos\phi ,y\to \;{\left(r^{2}+a^{2}\right)}^{1/2}sin\theta sin\phi ,z\to \;rcos\theta ,t\to \;t.$$

Here, *a* is the coordinate-transformation parameter. The metric (3) under the new coordinate system becomes

(5) $$ds^{2}=-dt^{2}+\frac{\Sigma }{r^{2}+a^{2}}dr^{2}+\Sigma d\theta^{2}+\left(r^{2}+a^{2}\right)sin^{2}\theta d\phi^{2},$$

where $\Sigma =r^{2}+a^{2}cos^{2}\theta$.

According to our previous research [14], [15], metric (5) describes an empty ellipsoid spacetime and can be rewritten in the following orthogonal form

(6) $$ds^{2}=-\frac{r^{2}+a^{2}}{\Sigma }{\left(dt-asin^{2}\theta d\phi \right)}^{2}+\frac{\Sigma }{r^{2}+a^{2}}dr^{2}+\Sigma d\theta^{2}\;\;+\frac{sin^{2}\theta }{\Sigma }{\left[(r^{2}+a^{2})d\phi -adt\right]}^{2}.$$

## 4 Derivation of an axisymmetric static solution: Kerr metric

To derive the Kerr metric, we may use the following ellipsoid orthogonal ansatz:

(7) $$ds^{2}=-\frac{f(r)}{\Sigma }{\left(dt-asin^{2}\theta d\phi \right)}^{2}+\frac{\Sigma }{g(r)}dr^{2}+\Sigma d\theta^{2}\;\;+\frac{sin^{2}\theta }{\Sigma }{\left[(r^{2}+a^{2})d\phi -adt\right]}^{2}.$$

Here, *f* and *g* are functions of *r*, and *a* is constant. Then the Kerr metric can be obtained directly from the ellipsoid symmetry. The metric tensor from the proposed ansatz (7) is given by

(8) $$g_{\mu \nu }=\left(\begin{array}{cccc} -\frac{f(r)-a^{2}sin^{2}\theta }{\Sigma } & 0 & 0\; & \;\frac{\left(f(r)-r^{2}-a^{2}\right)asin^{2}\theta }{\Sigma }\;\\ 0 & \frac{\Sigma }{g(r)} & \;0\; & 0\;\\ 0 & 0 & \Sigma \; & \;0\;\\ \frac{\left(f(r)-r^{2}-a^{2}\right)asin^{2}\theta }{\Sigma } & 0 & 0\; & \frac{\left[{\left(r^{2}+a^{2}\right)}^{2}-f(r)a^{2}sin^{2}\theta \right]sin^{2}\theta }{\Sigma }\; \end{array}\right).$$

Since the field equations are ten second-order partial differential equations with high nonlinearity, it is still too complicated to directly calculate the curvature tensor through two variables. Therefore, we may use energy conservation to cut a variable. Introducing energy parameter *E* and setting θ=0, we have

(9) $$E=\frac{f}{\Sigma }\overset{˙}{t}.$$

Lagrangian

(10) $$L=\frac{f}{\Sigma }{\overset{˙}{t}}^{2}-\frac{\Sigma }{g}{\overset{˙}{r}}^{2}$$

By substituting square of Eq. (9) into Eq. (10), and removing ${\overset{˙}{t}}^{2}$, we obtain

(11) $$L=\frac{f}{\Sigma }\left(\frac{E^{2}\Sigma^{2}}{f^{2}}\right)-\frac{\Sigma }{g}{\overset{˙}{r}}^{2}=\frac{E^{2}\Sigma }{f}-\frac{\Sigma }{g}{\overset{˙}{r}}^{2}.$$

Shift item

(12) $${\overset{˙}{r}}^{2}=\frac{g}{\Sigma }\left(\frac{\Sigma }{f}E^{2}-L\right)$$

When the photon moves along the r direction, since it has no mass, $\overset{¨}{r}=0$, L=0. We take the partial differential of (12) and obtain

(13) $$2\overset{˙}{r}\overset{¨}{r}=\overset{˙}{\left(\frac{g}{f}\right)}E^{2}=0.$$

Therefore g/f= constant. Let f=g; here, there is only one unknown variable f(r) left. We may now try to solve the Einstein field equations which are given by

(14) $$G_{ab}=R_{ab}-\frac{1}{2}g_{ab}R=8\pi T_{ab}.$$

For this purpose, we need to calculate the Christoffel symbols and the Ricci curvature tensor. The calculation methods are as follows

(15) $$\Gamma_{\mu \nu }^{\alpha }=\frac{1}{2}g^{\alpha \beta }\left(\partial_{\mu }g_{\nu \beta }+\partial_{\nu }g_{\beta \mu }-\partial_{\beta }g_{\mu \nu }\right),$$

(16) $$R_{\alpha \beta }=R_{\alpha \mu \beta }^{\mu }=\partial_{\rho }\Gamma_{\beta \alpha }^{\rho }-\partial_{\beta }\Gamma_{\rho \alpha }^{\rho }+\Gamma_{\rho \lambda }^{\rho }\Gamma_{\beta \alpha }^{\lambda }-\Gamma_{\beta \lambda }^{\rho }\Gamma_{\rho \alpha }^{\lambda }.$$

The non-zero terms of the Ricci tensors are given by

(17) $$R_{tt}=\frac{1}{2\Sigma^{3}f^{2}}\{\Sigma f^{3}f^{\prime \prime }+2f^{2}\left[-rf^{\prime }(a^{2}sin^{2}\theta +f\right)\;+f^{2}-2fa^{2}cos^{2}\theta +a^{2}sin^{2}\theta (r^{2}-a^{2})]\},$$

(18) $$R_{rr}=\frac{1}{2\Sigma f^{4}}\left[-f^{3}\Sigma f^{\prime \prime }+2f^{3}(a^{2}cos^{2}\theta +a^{2}+f^{\prime }r-f)\right],$$

(19) $$R_{\theta \theta }=\frac{-f^{\prime }r+r^{2}-a^{2}+f}{\Sigma },$$

(20) $$R_{\phi \phi }=\frac{-1}{2\Sigma^{3}f^{2}}\{-a^{2}f^{3}(-sin^{2}\theta \Sigma f^{\prime \prime }-2f^{2}((a^{2}sin^{2}\theta f+{\left(r^{2}+a^{2}\right)}^{2})rf^{\prime }-a^{2}sin^{2}\theta f^{2}+(-a^{4}cos^{4}\theta -2a^{2}r^{2}-r^{4})f+(a^{2}-r^{2}){\left(r^{2}+a^{2}\right)}^{2}\},$$

(21) $$R_{t\phi }=R_{\phi t}=\frac{asin^{2}\theta }{2\Sigma^{3}f^{2}}\{\Sigma f^{3}f^{\prime \prime }-2f^{2}[r(r^{2}+a^{2}+f)f^{\prime }-f^{2}\;\;+(a^{2}cos^{2}\theta -r^{2})f+a^{4}-r^{4}]\}.$$

There are second-order differential terms of *f* and trigonometric functions. Since there is only one variable left, we may use $R_{\theta \theta }=0$ to obtain the simplest first-order partial differential equation (22),

(22) $$-f^{\prime }r+r^{2}-a^{2}+f=0.$$

Equation (22) is solved by

(23) $$f(r)=r^{2}+C_{1}r+a^{2}.$$

Note that the Kerr metric is asymptotically flat, which means that f(r)/Σ will approach the Schwarzschild metric 1−2M/r at larger *r*. Therefore, in equation (23), $C_{1}$ is a mass function, that can be chosen as −2M. Finally, we obtain the Kerr metric via an ellipsoid coordinate transformation and an orthogonal metric ansatz:

(24) $$ds^{2}=-\frac{r^{2}-2Mr+a^{2}}{r^{2}+a^{2}cos^{2}\theta }{\left(dt-asin^{2}\theta d\phi \right)}^{2}+\frac{r^{2}+a^{2}cos^{2}\theta }{r^{2}-2Mr+a^{2}}+(r^{2}+a^{2}cos^{2}\theta )d\theta^{2}+\frac{sin^{2}\theta }{r^{2}+a^{2}cos^{2}\theta }{\left[(r^{2}+a^{2})d\phi -adt\right]}^{2}.$$

## 5 Generalization to Vaidya–Kerr black hole

To obtain a radiating Kerr black hole, the following coordinate transformations can be performed on the Kerr metric (24):

(25) $$dt=du^{\prime }+\frac{r^{2}+a^{2}}{\Delta_{K}}dr^{\prime },d\phi =d\phi^{\prime }+\frac{a}{\Delta_{K}}dr^{\prime },$$

where $\Delta_{K}=r^{2}-2Mr+a^{2}$. Then we give the Kerr metric in Eddington–Finkelstein retarded coordinates (u,r,θ,ϕ) as follows:

(26) $$ds^{2}=-Gdu^{2}-2dudr-2asin^{2}\theta (1-G)dud\phi +2asin^{2}\theta drd\phi +\Sigma d\theta^{2}+sin^{2}\theta \left[\Sigma -a^{2}sin^{2}\theta (G-2)\right]d\phi^{2}.$$

Here $G=1-\frac{2Mr}{\Sigma }$. We introduce a nonstatic mass m(u) to the Kerr spacetime and then obtain the Vaidya–Kerr metric as follows:

(27) $$ds^{2}=-\overset{¯}{G}du^{2}-2dudr-2asin^{2}\theta (1-\overset{¯}{G})dud\phi +2asin^{2}\theta drd\phi +\Sigma d\theta^{2}+sin^{2}\theta \left[\Sigma -a^{2}sin^{2}\theta (\overset{¯}{G}-2)\right]d\phi^{2}.$$

Here, $\overset{¯}{G}=1-\frac{2m(u)r}{\Sigma }$, and the delta function becomes $\Delta_{VK}=r^{2}-2m(u)r+a^{2}$. Now, after generalization, the covariant components of the metric tensor are expressed as matrix $g_{ab}$

(28) $$g_{ab}=\left(\begin{array}{cccc} -\overset{¯}{G} & -1 & 0\; & \;-asin^{2}\theta (1-\overset{¯}{G})\;\\ -1 & 0 & 0\; & \;asin^{2}\theta \;\\ 0 & 0 & \Sigma \; & \;0\;\\ -asin^{2}\theta (1-\overset{¯}{G}) & \;asin^{2}\theta & 0\; & sin^{2}\theta \left[\Sigma -a^{2}sin^{2}\theta (\overset{¯}{G}-2)\right]\; \end{array}\right).$$

This completes the radiating Kerr metric via ellipsoid coordinate transformation. Next we will discuss the physical properties of this metric (27).

## 6 Physical properties of the Vaidya–Kerr metric

### 6.1 Singularity

By considering the $g_{uu}$ component of the metric (27), it is clear that there is a coordinate singularity at $\Sigma =r^{2}+a^{2}cos^{2}\theta =0$, that is: r=0 and θ=π/2. To detect whether this is actually a curvature singularity exists in spacetime, we calculate the Ricci tensors $R_{ab}$ and list only the non-zero terms as follows:

(29) $$R_{uu}=\frac{-a^{2}rsin^{2}\theta \Sigma \overset{¨}{m}-2\overset{˙}{m}(a^{4}cos^{4}\theta -a^{4}cos^{2}\theta +a^{2}r^{2}+r^{4})}{\Sigma^{3}},$$

(30) $$R_{u\phi }=R_{\phi u}=\frac{\left[a^{2}rsin^{2}\theta \Sigma \overset{¨}{m}+\overset{˙}{m}(a^{4}cos^{4}\theta -2a^{4}cos^{2}\theta +2a^{2}r^{2}+3r^{4})\right]asin^{2}\theta }{\Sigma^{3}},$$

(31) $$R_{u\theta }=R_{\theta u}=\frac{-2\overset{˙}{m}ra^{2}cos\theta sin\theta }{\Sigma^{2}},$$

(32) $$R_{\theta \phi }=R_{\phi \theta }=\frac{2\overset{˙}{m}ra^{3}cos\theta \sin^{3}\theta }{\Sigma^{2}},$$

(33) $$R_{\phi \phi }=\frac{\left[-a^{2}rsin^{2}\theta \Sigma \overset{¨}{m}+2\overset{˙}{m}(a^{4}cos^{4}\theta -a^{2}r^{2}-2r^{4})\right]a^{2}sin^{4}\theta }{\Sigma^{3}}.$$

The Ricci scalar is $R=g^{ab}$ $R_{ab}$, the Ricci invariant is $R=R_{ab}$ $R^{ab}$, and the Kretschmann invariant is $K=R_{abcd}R^{abcd}$ (where $R_{abcd}$ is the Riemann tensor). For metric (27), they read

Ricci scalar R=0,

Ricci invariant R=0,

Kretschmann invariant

(34) $$K=\frac{48m{\left(u\right)}^{2}(-a^{6}cos^{6}\theta +15a^{4}r^{2}cos^{4}\theta -15a^{2}r^{4}cos^{2}\theta +r^{6})}{\Sigma^{6}}.$$

Now we see that for the radiating rotating black hole, Σ=0 is a scalar polynomial singularity, which is given by r=0 and θ=π/2. This set of points denotes a ring in the equatorial plane with a radius of *a*, similar to a stationary Kerr black hole. When m(u)=0, the metric (27) reduces to flat Minkowski spacetime. The Riemann tensor is identically zero: $R_{abcd}=0$.

### 6.2 Event horizon

To solve the event horizon from delta function, we have

(35) $$\Delta_{VK}=r^{2}+a^{2}-2m(u)r.$$

Let $\Delta_{VK}=(r-r_{+})(r-r_{-})=0$, we obtain the two roots of equation (35) as:

(36) $$r_{\pm }=m(u)\pm \sqrt{m{\left(u\right)}^{2}-a^{2}}.$$

Thus, on the surface $r=r_{+}$ and $r=r_{-}$, where $\Delta_{VK}=0$, and these surfaces are event horizons. In Kerr metric (24) using Boyer–Lindquist coordinates, $r=r_{+}$, and $r=r_{-}$, where $\Delta_{K}=0$ are event horizons and the coordinate singularity. For rotating black holes, this equation physically means that the rotation parameter, *a*, has a limitation of m(u). Therefore, $0\leq \frac{a}{m(u)}\leq 1$. To describe the spin of a black hole, a dimensionless spin parameter $a^{⁎}$ is introduced, and defined as $\frac{a}{m(u)}$ [22]. If all constants are restored, then we have

(37) $$a^{⁎}=\frac{a}{m(u)}=\left(\frac{J}{cm(u)}\right){\left(\frac{Gm(u)}{c^{2}}\right)}^{-1}=\frac{Jc}{Gm{\left(u\right)}^{2}}.$$

The range of $a^{⁎}$ is $0\leq a^{⁎}\leq 1$. Then, we rewrite the radius of the outer event horizon as

(38) $$r_{+}=\frac{Gm(u)}{c^{2}}\left(1+\sqrt{1-a^{{⁎}^{2}}}\right).$$

### 6.3 Ergosphere

In Vaidya spacetime (1) the horizon is also the surface where $g_{uu}$ change sign, in Vaidya–Kerr spacetime (27) these surfaces do not coincide. We have that

(39) $$g_{uu}=-1+\frac{2m(u)r}{\Sigma }=-\frac{1}{\Sigma }\left(r^{2}-2m(u)r+a^{2}cos^{2}\theta \right)=-\frac{1}{\Sigma }(r-r_{s+})(r-r_{s-})=0.$$

We obtain the two roots of equation (39) as:

(40) $$r_{s\pm }=m(u)\pm \sqrt{m{\left(u\right)}^{2}-a^{2}cos^{2}\theta }=\frac{Gm(u)}{c^{2}}\left(1\pm \sqrt{1-a^{{⁎}^{2}}cos^{2}\theta }\right).$$

Therefore, the region, i.e. $r_{+}<r<r_{s+}$, is called “ergoregion”, and its outer boundary $r=r_{s+}$ is called “ergosphere”. The ergosphere touches the event horizon at the axisymmetric pole, where θ=0, and θ=π.

### 6.4 Frame dragging and ZAMO

Let us consider an observer falls into the rotating black hole with zero angular momentum. Such an observer is named ZAMO, which stands for “zero angular momentum observer”. For the radiating Kerr metric (27), the trajectory of the ZAMO has a non-zero angular velocity given by

(41) $$\Omega =-\frac{g_{u\phi }}{g_{\phi \phi }}=\frac{2m(u)ar}{{\left(r^{2}+a^{2}\right)}^{2}-a^{2}\Delta_{VK}sin^{2}\theta }.$$

At $r=r_{+}$, $\Delta_{VK}=0$, the angular velocity at the event horizon is given by

(42) $$\Omega_{H}\equiv \frac{2m(u)ar_{+}}{{\left(r^{2}+a^{2}\right)}^{2}}=\frac{a}{2m(u)r_{+}}=\frac{a}{r_{+}^{2}+a^{2}}.$$

Notice that in equation (41)

(43) $${\left(r^{2}+a^{2}\right)}^{2}>a^{2}sin^{2}\theta (r^{2}+a^{2}-2m(u)r).$$

Therefore, we always have Ω/(m(u)a)>0: the angular velocity of the ZAMO has the same sign as the angular velocity m(u)a of the black hole, namely, the motion of ZAMO is co-rotating with the black hole. In other words, the ZAMO is dragged by the gravitational field of the black hole, acquiring an angular velocity co-rotating with the black hole. This is called *frame-dragging*, which is predicted by Einstein's general relativity and the predicted frame-dragging effect around a planet with the mass and size of our Earth, as measured by the Gravity Probe B gyroscopes, is very small [23].

## 7 Energy-momentum tensor

According to the definition of Einstein's field equations (14), the Einstein tensor is proportional to the energy-momentum tensor (EMT). Applying $g^{ab}$ to Einstein's field equations, we obtain

(44) $$g^{ab}\left(R_{ab}-\frac{1}{2}g_{ab}R\right)=8\pi g^{ab}T_{ab},R-\frac{1}{2}(4R)=8\pi T,R=-8\pi T.$$

Where *R* denotes the Ricci scalar. In metric (27), we have R=0. Therefore, T=0. Using the above results, we can easily check that this EMT satisfies energy conservation condition ${▽}_{b}T^{ab}=0$. Applying $g_{ab}$ to ${▽}_{b}T^{ab}$, we have

(45) $$g_{ab}({▽}_{b}T^{ab})={▽}_{b}(g_{ab}T^{ab})={▽}_{b}T=0.$$

Therefore, metric (27) is an exact solution of Einstein's field equations, and the Einstein tensor can be expressed as follows:

(46) $$G_{\mu \nu }=8\pi (T_{\mu \nu }^{N}+T_{\mu \nu }^{M}),$$

where $T_{\mu \nu }^{N}$ is the EMT of the null radiation, and $T_{\mu \nu }^{M}$ represents the EMT of the matter field. When we discuss the static solutions in general relativity, the source is the matter field. In this study we discuss the non-static solutions; therefore, the source is the null radiation and the matter field. The EMT of this metric (27) is given by

(47) $$T_{uu}=\frac{-a^{2}rsin^{2}\theta \Sigma \overset{¨}{m}-2\overset{˙}{m}(a^{4}cos^{4}\theta -a^{4}cos^{2}\theta +a^{2}r^{2}+r^{4})}{8\pi \Sigma^{3}},$$

(48) $$T_{u\phi }=T_{\phi u}=\frac{\left[a^{2}rsin^{2}\theta \Sigma \overset{¨}{m}+\overset{˙}{m}(a^{4}cos^{4}\theta -2a^{4}cos^{2}\theta +2a^{2}r^{2}+3r^{4})\right]asin^{2}\theta }{8\pi \Sigma^{3}},$$

(49) $$T_{u\theta }=T_{\theta u}=\frac{-2\overset{˙}{m}ra^{2}cos\theta sin\theta }{8\pi \Sigma^{2}},$$

(50) $$T_{\theta \phi }=T_{\phi \theta }=\frac{2\overset{˙}{m}ra^{3}cos\theta \sin^{3}\theta }{8\pi \Sigma^{2}},$$

(51) $$T_{\phi \phi }=\frac{\left[-a^{2}rsin^{2}\theta \Sigma \overset{¨}{m}+2\overset{˙}{m}(a^{4}cos^{4}\theta -a^{2}r^{2}-2r^{4})\right]a^{2}sin^{4}\theta }{8\pi \Sigma^{3}}.$$

When the rotation parameter *a* vanishes, metric (27) recoveries to the Vaidya metric, and the EMT returns to $T_{\mu \nu }^{N}=T_{uu}=-\frac{\overset{˙}{m}}{4\pi r^{2}}$. According to Petrov classification [24], the Vaidya–Kerr black-hole metric (27) is classified as Petrov type II with a twisting, shear-free, null congruence, just as is a Kerr black hole. However, it is classified as Petrov type D for a Kerr black hole.

## 8 Results and discussion

When mass function m(u) is replaced by a constant *M*, the Vaidya–Kerr metric (27) returns to the Kerr metric in Eddington–Finkelstein coordinates. According to Erbin [25], we may turn it to Boyer–Lindquist coordinates (t,r,θ,ϕ) through appropriate transformations. Therefore, metric (27) is a nonstationary generalization of the Kerr metric. Furthermore, when rotation parameter *a* vanishes, metric (27) recovers to a Vaidya retarded metric (1), so metric (27) is also an axisymmetric generalization of the Vaidya metric. Next, we will discuss the surface area of event horizon, surface gravity and Hawking radiation of the Vaidya–Kerr black hole from a thermodynamic point of view. Note that we treat the rotation parameter *a* as a constant, not a variable.

### 8.1 Surface area of event horizon and black-hole entropy

The event-horizon surface area of a Kerr black hole is given by the following equation:

(52) $$A=\int_{0}^{2\pi }d\theta \int_{0}^{\pi }d\phi sin\phi (r_{+}^{2}+a^{2})=4\pi (r_{+}^{2}+a^{2}).$$

By substituting equation (38) into (52), we obtain the formula for the horizon surface area of the Vaidya–Kerr black hole as follows:

(53) $$A=8\pi {\left(\frac{Gm(u)}{c^{2}}\right)}^{2}\left(1+\sqrt{1-a^{{⁎}^{2}}}\right).$$

The Bekenstein–Hawking formula [26], [27] shows that black-hole entropy is proportional to the event horizon's surface area; thus, using this and equation (53), we obtain the following equation:

(54) $$S_{BH}=\frac{k_{b}c^{3}A}{4G\hbar }=\frac{2\pi Gk_{b}}{\hbar c}m{\left(u\right)}^{2}\left(1+\sqrt{1-a^{{⁎}^{2}}}\right),$$

where $k_{b}$ denotes the Stefan–Boltzmann constant.

The mass of a radiating rotating black hole decreases with time, leading to a decrease in the surface area and entropy. This violates the second law of thermodynamics. Bekenstein proposed a generalized second law of thermodynamics to solve this problem [28], by which the entropy of a system never decreases with time, i.e.,

(55) $$\delta S=\left(S+S_{BH}\right)\geq 0.$$

According to the generalized second law of thermodynamics, the black-hole entropy, $S_{BH}$, can be transformed into other forms, and the total entropy of the universe never decreases with time.

### 8.2 Surface gravity and Hawking radiation

On the killing horizon, the surface gravity of the Kerr metric expressed as the inner and outer horizon radii [29] is

(56) $$\kappa =\frac{r_{+}-r_{-}}{2(r_{+}^{2}+a^{2})}.$$

By substituting equation (36) into (56), the surface gravity of Vaidya–Kerr metric (27) on the Killing horizon is given by

(57) $$\kappa =\frac{\sqrt{m{\left(u\right)}^{2}-a^{2}}}{2m(u)\left(m(u)+\sqrt{m{\left(u\right)}^{2}-a^{2}}\right)}.$$

Hawking proved that the thermal radiation emitted by black holes has an absolute temperature, i.e., the Hawking temperature, $T_{H}$. Hawking temperature is well known to be proportional to the surface gravity on the horizon [30]; therefore, the Hawking temperature of the Vaidya–Kerr black hole can be obtained as follows:

(58) $$T_{H}=\frac{\kappa }{2\pi }=\frac{\sqrt{m{\left(u\right)}^{2}-a^{2}}}{4\pi m(u)\left(m(u)+\sqrt{m{\left(u\right)}^{2}-a^{2}}\right)}.$$

By restoring all constants, we have

(59) $$T_{H}=\frac{\hbar c^{3}}{4\pi k_{b}Gm(u)}\frac{\sqrt{1-a^{{⁎}^{2}}}}{\left(1+\sqrt{1-a^{{⁎}^{2}}}\right)}.$$

From equation (59), $T_{H}$ is zero when the black hole has maximum spin $a^{⁎}=1$. The Hawking temperature cannot be absolute zero according to the third law of thermodynamics [28]. When $a^{⁎}=0$, we recover the Hawking temperature of the Vaidya metric as

(60) $$T_{H}=\frac{\hbar c^{3}}{8\pi k_{b}Gm(u)}.$$

Note that the ratio of $T_{H}$ under maximum rotation $\left(a^{⁎}=1\right)$ to $T_{H}$ that under minimum rotation $\left(a^{⁎}=0\right)$ is only 1/2. Thus, $a^{⁎}$ does not have a major role in determining $T_{H}$. The mass function m(u), which is inversely proportional to Hawking radiation, plays the major role in determining $T_{H}$. To estimate the value of $T_{H}$, we choose black holes with the angular momentum and the mass of Pluto ([31]) and the Sun ([32]), as well as the supermassive black hole in the core of the M87 galaxy newly investigated by the Event Horizon Telescope. This is the first direct astronomical observation of black-hole horizon data [33]. We choose the Pluto-mass black hole as a testing object because according to Hawking's prediction, primordial black holes in the early universe did not form from stellar gravitational collapse, so their masses can be far below stellar mass [34].

Table 1 lists the calculated results using equations (38) and (60). We obtain the $T_{H}$ values of the Pluto-mass, sun-mass, and M87 black holes as 9.42 K, $6.08\times 10^{-8}$ K, and $8.78\times 10^{-18}$ K, respectively. The $T_{H}$ values of the sun and the M87 black hole are much smaller than the 3-K cosmic microwave background (CMB) radiation; therefore, they cannot be measured astronomically. Only the $T_{H}$ of a rotating Pluto-mass black hole is slightly greater than the CMB. We therefore need tools with sufficient resolution to detect subtle fluctuations in the CMB.

**Table 1:** Physical parameters of Pluto, the sun and the M87 supermassive rotating black hole.

Physical parameters		Pluto	sun	M87 black hole
*m*: mass	[kg]	1.30 × 10^22^	1.99 × 10^30^	1.29 × 10^40^
*R*: radius	[m]	1.74 × 10^6^	6.95 × 10^8^	3.70 × 10^15^
*r*_+_: outer event horizon radius	[m]	1.93 × 10^−5^	2.92 × 10^3^	1.92 × 10^13^
*J*: angular momentum	[kg⋅m^2^/sec]	1.79 × 10^29^	1.92 × 10^41^	3.34 × 10^61^
*a*: rotation parameter	[m]	4.58 × 10^−2^	3.22 × 10^2^	8.63 × 10^12^
*a*⁎: spin parameter		8.00 × 10^−1^	2.18 × 10^−1^	9.00 × 10^−1^
*T*_*H*_: Hawking temperature	[K]	9.42	6.08 × 10^−8^	8.78 × 10^−18^

## 9 Conclusion

This research derives a radiating rotating black-hole solution via ellipsoid coordinate transformation. This radiating Kerr metric, the Vaidya–Kerr metric, is an axisymmetric generalization of the Vaidya metric. The study results demonstrate that the energy momentum tensor has the form of a Petrov type II fluid. The inner and outer horizon radii, the ergosphere radii, as well as the angular velocity at the event horizon are obtained, and the event horizon's surface area, black-hole entropy, surface gravity, and Hawking radiation are derived and expressed by the mass function and spin parameter. Results indicated that the Hawking temperature of a rotating solar-mass black hole is much greater than that of the M87 supermassive rotating black hole. However, both values are considerably too small to be measured at present. Only the rotating Pluto-mass black hole, which might theoretically have been formed in the early universe, has a Hawking temperature slightly greater than 3-K CMB radiation, which would allow its value to be potentially detected by high-resolution tools. The derivation method in this study is concise and can be further generalized to other non-static solutions by replacing the mass function with non-static ones, which deserve further study in the future.

## Declarations

### Author contribution statement

Yu-Ching Chou: Conceived and designed the analysis; Analyzed and interpreted the data; Contributed analysis tools or data; Wrote the paper.

### Funding statement

This research did not receive any specific grant from funding agencies in the public, commercial, or not-for-profit sectors.

### Competing interest statement

The authors declare no conflict of interest.

### Additional information

No additional information is available for this paper.

## References

[1] Schwarzschild K. (1916). Über das gravitationsfeld eines massenpunktes nach der einsteinschen theorie.

[2] Reissner H. (1916). Über die eigengravitation des elektrischen feldes nach der einsteinschen theorie. Ann. Phys..

[3] Nordström G. (1918). On the energy of the gravitation field in Einstein's theory. Proc. K. Ned. Akad. Wet., Ser. B, Phys. Sci..

[4] Kerr R.P. (1963). Gravitational field of a spinning mass as an example of algebraically special metrics. Phys. Rev. Lett..

[5] Newman E.T., Couch E., Chinnapared K., Exton A., Prakash A., Torrence R. (1965). Metric of a rotating, charged mass. J. Math. Phys..

[6] Vaidya P. (1943). The external field of a radiating star in general relativity. Curr. Sci..

[7] Hawking S. (1974). Black-hole evaporation. Nature.

[8] Newman E.T., Adamo T. (2014). Kerr–Newman metric. Scholarpedia.

[9] Brauer O., Camargo H., Socolovsky M. (2015). Newman–Janis algorithm revisited. Int. J. Theor. Phys..

[10] Penrose R., Rindler W. (1984). Spinors and Space-Time: Volume 1, Two-Spinor Calculus and Relativistic Fields, Vol. 1.

[11] Nerozzi A. A new approach to the Newman–Penrose formalism. arxiv:1109.4400.

[12] Meinel R. (2016). A physical derivation of the Kerr–Newman black hole solution. 1st Karl Schwarzschild Meeting on Gravitational Physics.

[13] Kramer D. (1987). The Ernst equation in general relativity. Czechoslov. J. Phys. B.

[14] Chou Y.-C. (2017). A derivation of the Kerr metric by ellipsoid coordinate transformation. Int. J. Phys. Sci..

[15] Chou Y.-C. (2018). A derivation of the Kerr–Newman metric using ellipsoid coordinate transformation. J. Appl. Phys. Sci. Int..

[16] Ibohal N. (2005). Rotating metrics admitting non-perfect fluids. Gen. Relativ. Gravit..

[17] Ghosh S.G., Maharaj S.D. (2015). Radiating Kerr-like regular black hole. Eur. Phys. J. C.

[18] Eddington A.S. (1924). A comparison of Whitehead's and Einstein's formula. Nature.

[19] Finkelstein D. (1958). Past-future asymmetry of the gravitational field of a point particle. Phys. Rev..

[20] Boehmer C.G., Hogan P.A. (2017). A Vaidya-type generalization of Kerr spacetime. Mod. Phys. Lett. A.

[21] Taub A. (1976). High frequency gravitational radiation in Kerr–Schild space-times. Commun. Math. Phys..

[22] Reynolds C. (2013). The spin of supermassive black holes. Class. Quantum Gravity.

[23] Everitt C.F., DeBra D., Parkinson B., Turneaure J., Conklin J., Heifetz M., Keiser G., Silbergleit A., Holmes T., Kolodziejczak J. (2011). Gravity probe B: final results of a space experiment to test general relativity. Phys. Rev. Lett..

[24] Petrov A.Z. (2000). The classification of spaces defining gravitational fields. Gen. Relativ. Gravit..

[25] Erbin H. (2015). Janis–Newman algorithm: simplifications and gauge field transformation. Gen. Relativ. Gravit..

[26] Bekenstein J.D. (1973). Black holes and entropy. Phys. Rev. D.

[27] Hawking S.W. (1975). Particle creation by black holes. Commun. Math. Phys..

[28] Bardeen J.M., Carter B., Hawking S.W. (1973). The four laws of black hole mechanics. Commun. Math. Phys..

[29] Raine D.J., Thomas E.G. (2010). Black Holes: An Introduction.

[30] Page D.N. (2005). Hawking radiation and black hole thermodynamics. New J. Phys..

[31] Williams D. (2015). Pluto fact sheet, NASA. https://nssdc.gsfc.nasa.gov/planetary/factsheet/plutofact.html.

[32] Iorio L. (2012). Constraints on the location of a putative distant massive body in the solar system from recent planetary data. Celest. Mech. Dyn. Astron..

[33] Faber A., Akiyama K., Alberdi A., Alef W., Asada K., Azulay R., Baczko A.-K., Ball D., Baloković M., Barrett J. (2019). First M87 event horizon telescope results. I. The shadow of the supermassive black hole. Astrophys. J. Lett..

[34] Hawking S. (1971). Gravitationally collapsed objects of very low mass. Mon. Not. R. Astron. Soc..

